# The Impact of Photoperiod on the Leptin Sensitivity and Course of Inflammation in the Anterior Pituitary

**DOI:** 10.3390/ijms21114153

**Published:** 2020-06-10

**Authors:** Maciej Wójcik, Andrzej Przemysław Herman, Dorota Anna Zieba, Agata Krawczyńska

**Affiliations:** 1 The Kielanowski Institute of Animal Physiology and Nutrition, Polish Academy of Sciences, ul. Instytucka 3, 05-110 Jabłonna, Poland; a.herman@ifzz.pl (A.P.H.); a.krawczynska@ifzz.pl (A.K.); 2 Laboratory of Biotechnology and Genomics, Department of Nutrition, Animal Biotechnology and Fisheries, Agricultural University of Krakow, 30-248 Krakow, Poland; dorota.zieba-przybylska@urk.edu.pl

**Keywords:** leptin, leptin resistance, IL1β, IL6, TNFα, pituitary, sheep, LPS, photoperiod, relative gene expression

## Abstract

Leptin has a modulatory impact on the course of inflammation, affecting the expression of proinflammatory cytokines and their receptors. Pathophysiological leptin resistance identified in humans occurs typically in sheep during the long-day photoperiod. This study aimed to determine the effect of the photoperiod with relation to the leptin-modulating action on the expression of the proinflammatory cytokines and their receptors in the anterior pituitary under physiological or acute inflammation. Two in vivo experiments were conducted on 24 blackface sheep per experiment in different photoperiods. The real-time PCR analysis for the expression of the genes *IL1B*, *IL1R1*, *IL1R2*, *IL6*, *IL6R*, *IL6ST*, *TNF*, *TNFR1,* and *TNFR2* was performed. Expression of all examined genes, except *IL1β* and *IL1R2*, was higher during short days. The leptin injection increased the expression of all examined genes during short days. In short days the synergistic effect of lipopolysaccharide and leptin increased the expression of *IL1B*, *IL1R1*, *IL1R2*, *IL6*, *TNF,* and *TNFR2,* and decreased expression of *IL6ST*. This mechanism was inhibited during long days for the expression of *IL1R1, IL6, IL6ST,* and *TNFR1.* The obtained results suggest the occurrence of leptin resistance during long days and suggest that leptin modulates the course of inflammation in a photoperiod-dependent manner in the anterior pituitary.

## 1. Introduction

Leptin is a pleiotropic hormone that is secreted mainly by white adipose tissue, and its concentration is correlated with Body Mass Index (BMI) [1]. Leptin plays an essential role in the regulation of hypothalamic–pituitary endocrine pathways. It is widely expressed in the anterior pituitary (AP) cells, which are leptin positive in the following proportions: 70% of adrenocorticotropic hormone (ACTH) cells, 21% of growth hormone (GH) cells, 29% of luteinizing hormone (LH) cells, 33% of follicle-stimulating hormone (FSH) cells, 32% of thyroid-stimulating hormone (TSH) cells, and 64% folliculo-stellate cells, whereas very few prolactin (PRL) cells were positive (3%) [2]. Moreover, all of the AP cell types express the leptin receptor [3], which may confirm the significant effect of leptin on gonadotropin secretion in the pituitary [4]. Studies have shown that *ob/ob* mice are infertile because of disturbances in the secretion of gonadotropins, which leads to atrophy of the reproductive organs [5]. Moreover, in laboratory animals, treatment with leptin restored the reproductive functions by increasing the secretion of gonadotropins [4,6,7,8,9]. Furthermore, the mutation of the leptin receptor in humans leads to disturbances in body weight, sexual maturation, and secretion of growth and thyrotrophic hormones [10]. It is worth mentioning that, besides white adipose tissue, the expression of leptin was also confirmed in many tissues and organs, including AP [2]. After the discovery of the expression of leptin in the pituitary, it was thought that pituitary-produced leptin acts in an endocrine way, but currently, it is believed that leptin, locally synthesized in the pituitary, acts mostly in an auto- or paracrine manner [2]. Its signal is transduced by leptin receptors, which appear in large numbers at the pituitary level, including the longest, fully functional isoform OB-Rb [11,12]. However, the signal transduced by OB-Rb sometimes becomes inhibited, and therefore the sensitivity to leptin decreases, which is called leptin resistance (LEPres). Moreover, LEPres is characterized by the insensitivity of tissues and glands to this adipokine action and is associated with hyperphagia, reduced energy expenditure, and hyperleptinemia. The LEPres is observed in pathological conditions in humans, e.g., in obesity [13,14], and physiologically in photoperiodic animals, e.g., sheep [15,16,17]. Photoperiodism, as a seasonal environmental cycle, is a part of an interdisciplinary area in biology called chronobiology. In the photoperiodic animals, the seasonal rhythms reflected in changes in metabolism, nutritional behavior or reproductive status are regulated by the melatonin hormone [18], which is known as the “mother hormone of chronobiology”. In sheep, as was stated, the melatonin plasma concentration reaches the highest level during the short-day photoperiod [19,20]. During the short-day photoperiod, the duration of melatonin secretion is longer than during the long days (10–12 h vs. 8–10 h, respectively) [19]. As was shown in the research conducted on rats, lack of melatonin may cause LEPres [21]. Moreover, as it was shown, melatonin regulates the activity of the immune system and participates in the course of inflammation [22,23]. The close relationship was also observed between day length and plasma leptin levels in sheep. During a short day, the plasma leptin concentration remains low (2 ng/mL), while during a long day, it increases more than 3-fold (7 ng/mL) [24]. Furthermore, the increased leptin level during this period is accompanied by increased daily food intake and weight gain. The observed relationship remains in contrast with the physiological interactions of leptin, associated with a decrease in feed intake and weight reduction [25]. Chilliard et al., 2005 [26] stated that “the long-day sheep” is a suitable model for the study of hyperleptinemia. In turn, Zieba et al., 2008 proposed “the long day-sheep” as an animal model for obesity research due to its hyperphagia, decreased energy expenditure, and hyperleptinemia [17,27]. Photoperiod substantially controls several hormones released in an organism, and thus affects many physiologic and pathophysiologic processes in an organism, such as inflammation/immune response [28], reproduction/sexual maturation [29], metabolism [30], food intake [31], obesity/diabetes [32], cardiovascular diseases [33], cancer [34] and neurological disorders [35]. Most probably, variable sensitivity to leptin is related to the adaptation of these animals to annual changes in energy supply and demand [15]. Varying sensitivity of the central nervous system to peripheral hormones, such as leptin [16], ghrelin and orexin [36], is widely investigated. The mechanism of both physiological and pathological LEPres is still not fully understood. It is said that LEPres can be connected with decreased leptin transport across the blood–brain barrier [37], decreased leptin receptor expression, or defects in its intracellular signaling [38], but, as was shown in sheep, a suppressor of cytokine signaling (SOCS) 3 may also participate in the LEPres induction [17]. The occurrence of the LEPres at the pituitary level, in terms of the influence on the proinflammatory cytokines, is associated with the regulation of the hormonal activity of the hypothalamic–pituitary axes. Leptin affects the activity of the hypothalamic–pituitary–thyroid [39], somatotropic [40], adrenal [41], gonadal [42] and prolactin [43] axes. The tissue’s affinity for LEPres also requires further explanation.

Besides acting as a hormone regulating appetite, leptin exerts cytokine-like properties. It was found that sheep are a useful model for immunological studies [44], which can be used in leptin-related research because leptin also plays a role in the activation of the immune system, and is a mediator of inflammation. It is generally accepted that leptin is a proinflammatory adipokine [45], and, like other cytokines, may modulate the transduction of signals of different cytokines, affecting both cytokines and the expression of their receptors [46]. Leptin belongs to the long-chain helical cytokines and shows structural similarity to interleukins (IL) 2, 3, 6, 12, 15, granulocyte colony-stimulating factor, oncostatin M, prolactin or the growth hormone [47,48,49]. The role of leptin in inflammatory diseases is widely studied. Its proinflammatory influence has been demonstrated in autoimmune diseases, such as experimental autoimmune encephalomyelitis, inflammatory bowel disease, autoimmune diabetes, experimentally induced hepatitis, colitis, and glomerulonephritis [50,51]. Due to its elevated level, the leptin role was also studied in non-autoimmune diseases, such as atherosclerosis, type 2 diabetes, and chronic pulmonary inflammatory disease. As stated, most of the leptin’s effects in these diseases are related to its ability to induce a strong and sustained inflammatory response [51,52].

It was found that leptin dose-dependently stimulates the production of proinflammatory cytokines via monocytes, such as tumor necrosis factor (TNF)-α and IL6 [53], but does not have a direct influence on the expression or secretion of IL1β [54]. Interestingly, it was stated that leptin increased the expression of IL1β in many regions of the brain [55]. On the other side, the increased concentration of IL1β [56] and TNFα [57] enhanced expression of leptin, whereas for IL6, no similar effect of leptin was observed [56]. The mentioned results, in conclusion, indicate that leptin is involved in inflammation in both an auto- and paracrine manner [58]. Furthermore, the mentioned proinflammatory cytokines, including leptin, also influence the pituitary hormonal activity. The action of cytokines in the pituitary influences the production and release of hormones such as gonadotropins [59], the proliferation and survival of hormone-producing cells, tissue modeling, chemotaxis of immune cells infiltrating organs, tissue destruction and development of fibrosis [46]. Research on the rat primary cultures of AP cells showed that TNF inhibits the secretion of pituitary hormones ACTH, GH, LH and PRL in response to hypothalamic stimulation [60], while it was found that IL6 stimulates the secretion of ACTH, GH, PRL, LH and FSH [61,62,63]. These data underline the significant influence of cytokines on pituitary endocrine activity, and thus the significant impact of the LEPres in this approach.

Moreover, it was stated that IL1β downregulates the activity of the hypothalamic–pituitary–gonadal [59], –prolactin [61] and –thyroid [64] axes, and stimulates the hypothalamic–pituitary–adrenal [63] and –somatotropic [65] axes. The examined proinflammatory cytokines have pleiotropic effects on mammalian organisms. Knowing that these cytokines may disturb the secretion activity of the AP, it is worth noting that these cytokines reach this organ not only via the bloodstream, but are also directly synthesized by cells of this gland [66]. Due to the significant role of cytokines in pituitary hormones secretion, the analysis of genes encoding IL1β, IL6, and TNFα cytokines expression was performed. The experiment was carried out on sheep as a LEPres animal model and to verify its occurrence at the pituitary level. Therefore, results obtained for the long-day, LEPres sheep were compared with those obtained during the short-day photoperiod, to verify the role of leptin and day length in the course of inflammation. Furthermore, to analyze cytokines’ signaling pathways, and due to their diverse effects on signal transduction, the expression of genes encoding cytokines main receptors and the signal transducer was also examined.

Thus, the present study aimed to examine the influence of leptin on the induction and the course of inflammation in the sheep pituitary, depending on the photoperiodical conditions determining the occurrence of natural LEPres existing during a long day in this species.

## 2. Results

### 2.1. Gene Expression of Interleukin-1β and Its Receptors

**Figure 1:**
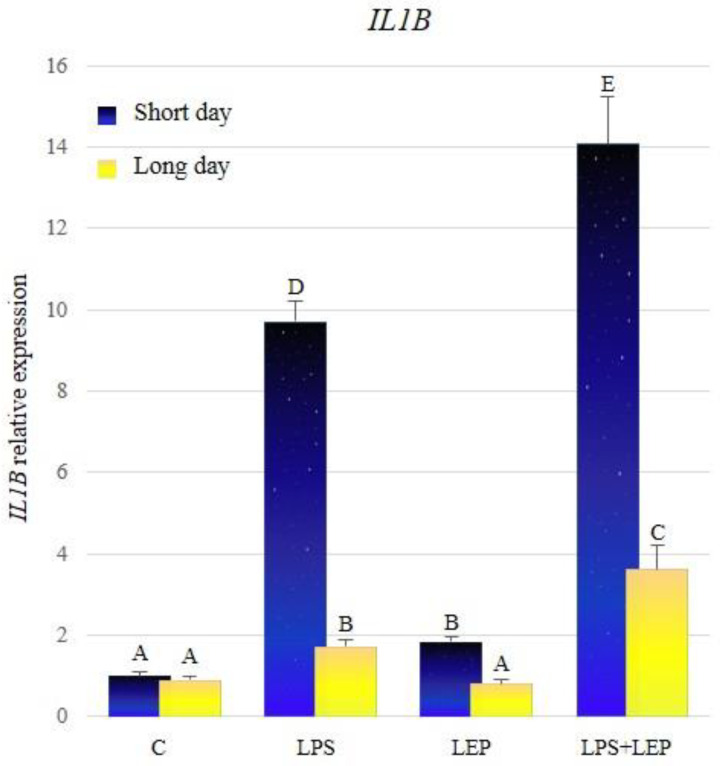
*IL1B* gene expression in relation to the mean of *HDAC* and *ACTB* reference genes in the sheep’s anterior pituitary during the short-day and long-day photoperiod. Groups: C—control groups, LPS—LPS-treated groups, LEP—leptin-treated groups, LPS+LEP—groups treated with LPS and leptin. The data are presented as means ± standard error of the mean (SEM). Letters indicate significant differences between research groups, according to post-hoc Fisher’s test.

Comparing the relative expression of the *IL1B* in control groups between different seasons, there was no significant difference stated (Figure 1, Table 1). Groups treated with lipopolysaccharide (LPS) showed a significant increase in the *IL1B* expression during both photoperiods, in relation to the corresponding control groups. The expression of the examined gene was significantly increased in the group treated with leptin, in comparison to the control group, but only during the short-day photoperiod. The leptin injection after prior LPS treatment caused higher *IL1B* gene expression, in comparison to groups treated only with LPS or LEP, regardless of the examined season. Moreover, the synergistic effect of both factors was revealed during both seasons. Comparing the *IL1B* expression between seasons, the gene expression increased much more significantly in the research groups during the short-day photoperiod.

**Figure 2:**
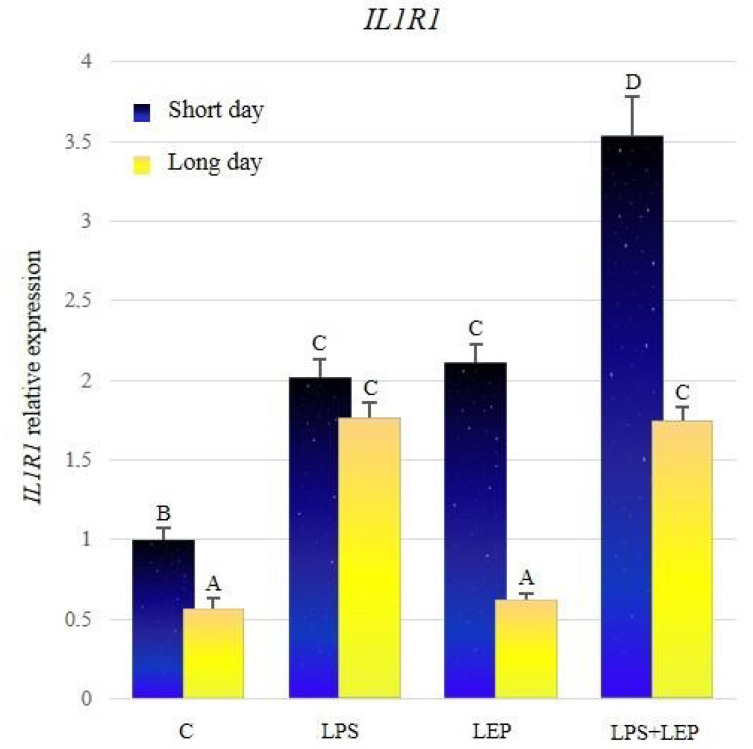
*IL1R1* gene expression in relation to the mean of *HDAC* and *ACTB* reference genes in the sheep’s anterior pituitary during the short-day and long-day photoperiod. Groups: C—control groups, LPS—LPS-treated groups, LEP—leptin-treated groups, LPS+LEP—groups treated with LPS and leptin. The data are presented as means ± standard error of the mean (SEM). Letters indicate significant differences between research groups, according to Fisher’s post-hoc test.

The significantly higher expression of *IL1R1* was stated in the control group from the short-day photoperiod, compared to the long-day photoperiod control group (Figure 2, Table 2). The LPS treatment significantly increased the examined gene’s expression compared to the corresponding control group during both photoperiods. Leptin injection stimulated the expression of *IL1R1* only during the short-day photoperiod, and the gene expression reached the level of groups stimulated with the LPS. In the short-day photoperiod, the expression of the examined gene in the LPS+LEP group was higher in comparison to the LPS- or leptin-treated group. In the long-day photoperiod, the *IL1R1* expression level in the LPS+LEP group did not differ from the one in the LPS-treated group. During both photoperiods, the gene expression in the LPS+LEP groups was increased in comparison to the corresponding control groups; however, only during the short-day photoperiod the effect of the LPS and leptin interaction was synergistic. In long-day photoperiod, the gene expression in the LSP + LEP group did not differ from that of the LPS group.

**Figure 3:**
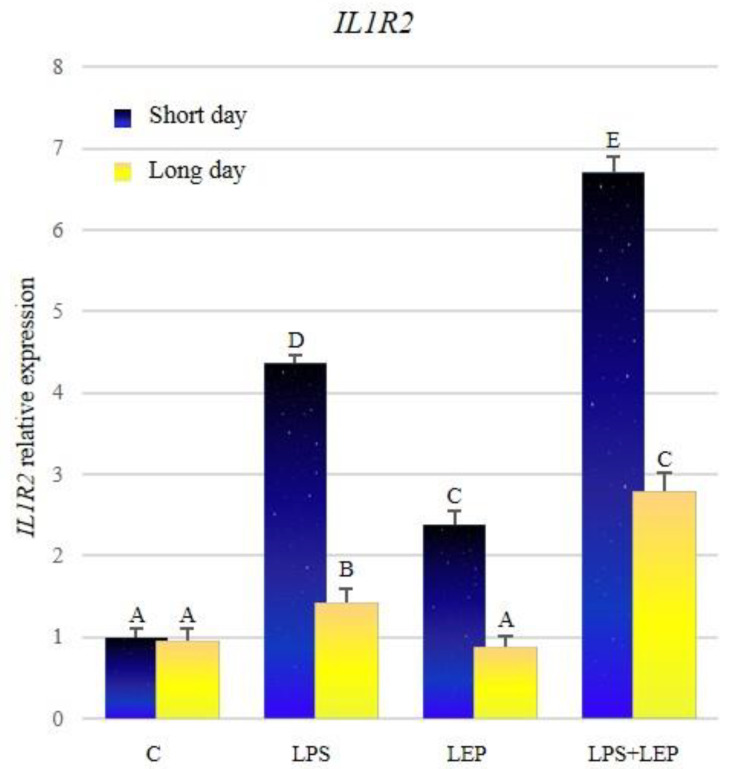
*IL1R2* gene expression in relation to the mean of *HDAC* and *ACTB* reference genes in the sheep’s anterior pituitary during the short-day and long-day photoperiod. Groups: C—control groups, LPS—LPS-treated groups, LEP—leptin-treated groups, LPS+LEP—groups treated with LPS and leptin. The data are presented as means ± standard error of the mean (SEM). Letters indicate significant differences between research groups, according to Fisher’s post-hoc test.

There was no significant difference between control groups from both photoperiods (Figure 3, Table 3). Both groups treated with the LPS showed a significant increase in the expression of *IL1R2*; however, the expression was significantly higher during the short-day photoperiod. Leptin injection stimulated the expression of the examined gene only during the short-day photoperiod. The synergistic effect of both research factors was observed during both photoperiods; however, the expression of the examined gene was higher during the short-day photoperiod. All research groups, except the controls, showed higher expressions during the short-day photoperiod.

### 2.2. Gene Expression of Interleukin-6, Its Receptor and Signal Transducer

**Figure 4:**
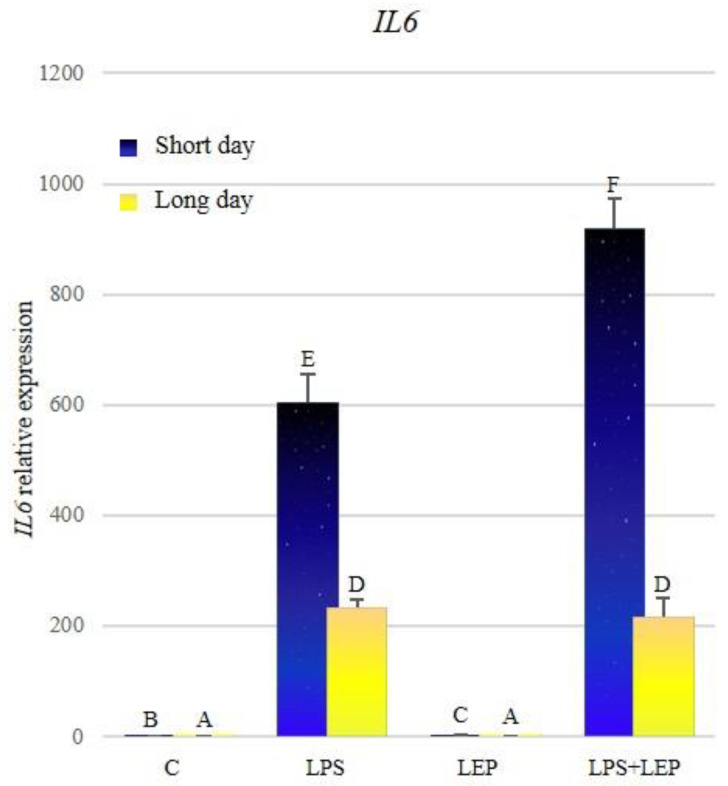
*IL6* gene expression in relation to the mean of *HDAC* and *ACTB* reference genes in the sheep’s anterior pituitary during the short-day and long-day photoperiod. Groups: C—control groups, LPS—LPS-treated groups, LEP—leptin-treated groups, LPS+LEP—groups treated with LPS and leptin. The data are presented as means ± standard error of the mean (SEM). Letters indicate significant differences between research groups, according to Fisher’s post-hoc test.

A significantly higher expression of *IL6* during the short-day photoperiod was stated (Figure 4, Table 4). During both photoperiods, treatment with LPS caused a significant increase in the examined gene’s expression; however, during the short-day photoperiod, this expression was higher. Leptin injection caused an increase in the expression only during the short-day photoperiod. The synergistic effect of examined factors on the expression was stated only during the short-day photoperiod. In the long-day photoperiod, the expression of *IL6* in the group treated with both factors did not differ significantly from the LPS-treated group. Moreover, in all groups, the expression of the examined gene was higher during the short-day photoperiod, in comparison to the corresponding groups in the long-day photoperiod.

**Figure 5:**
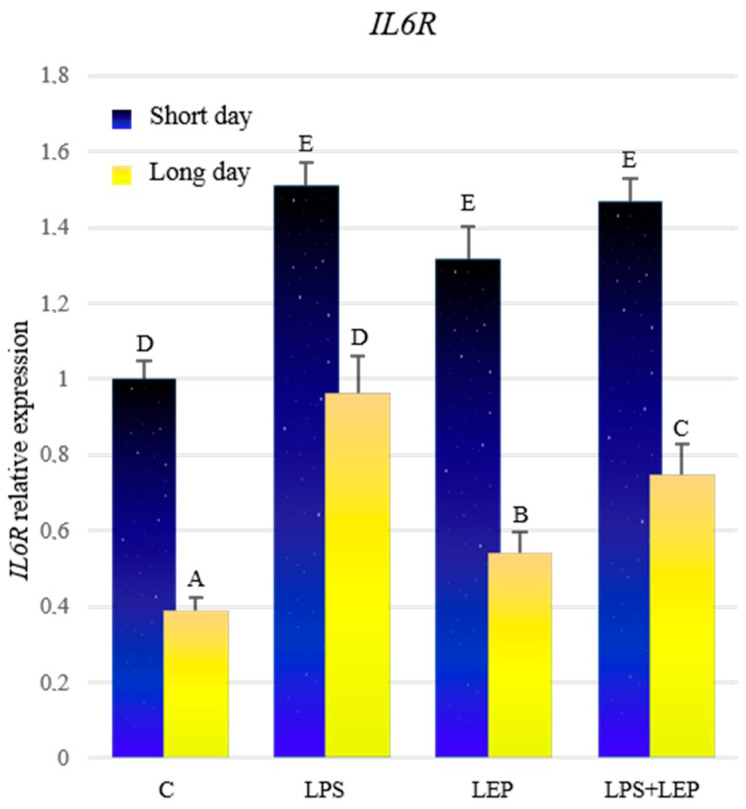
*IL6R* gene expression in relation to the mean of *HDAC* and *ACTB* reference genes in the sheep’s anterior pituitary during the short-day and long-day photoperiod. Groups: C—control groups, LPS—LPS-treated groups, LEP—leptin-treated groups, LPS+LEP—groups treated with LPS and leptin. The data are presented as means ± standard error of the mean (SEM). Letters indicate significant differences between research groups, according to Fisher’s post-hoc test.

Comparing the control groups, the expression of *IL6R* was significantly higher during the short-day photoperiod (Figure 5, Table 5). Treatment with LPS caused a significant increase in the expression of the examined gene in both photoperiods, in comparison to the corresponding control groups; however, in the short-day photoperiod, this expression was higher. Similarly, after the leptin treatment, the expression increased significantly during both photoperiods, in comparison to the corresponding control groups, but the expression was higher in the short-day photoperiod. Further, in the groups treated with both experimental factors, a significant increase in the expression was stated compared to the corresponding control groups. However, during the short-day photoperiod, the expression in the LPS+LEP group did not differ from that in the LPS- or leptin-treated groups, while during the long-day photoperiod, this expression was lower compared to the LPS group, and higher compared to the leptin-treated group. All groups from the short-day photoperiod showed a higher expression of the *IL6R* compared to the corresponding long-day photoperiod groups.

**Figure 6:**
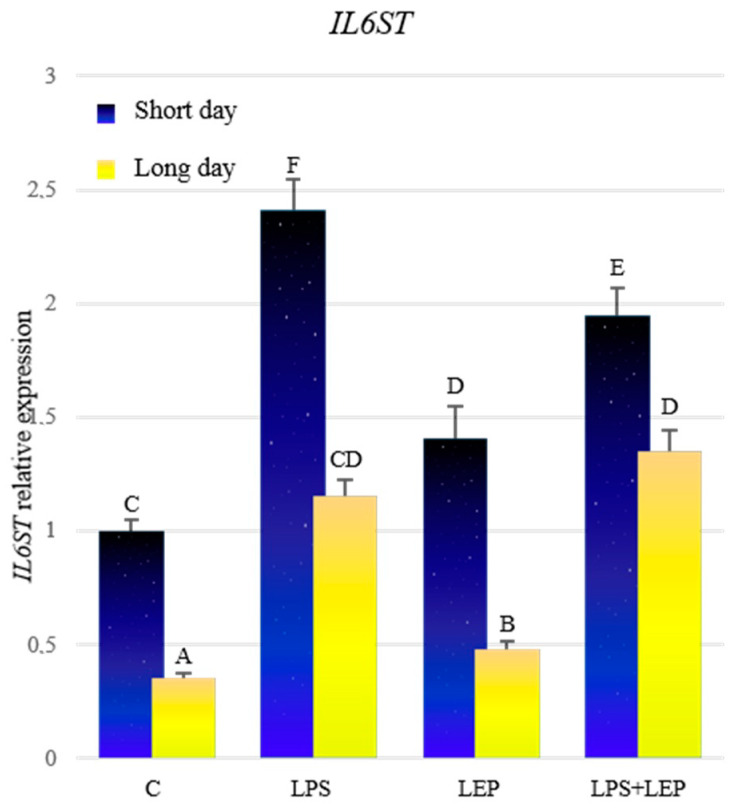
*IL6ST* gene expression in relation to the mean of *HDAC* and *ACTB* reference genes in the sheep’s anterior pituitary during the short-day and long-day photoperiod. Groups: C—control groups, LPS—LPS-treated groups, LEP—leptin-treated groups, LPS+LEP—groups treated with LPS and leptin. The data are presented as means ± standard error of the mean (SEM). Letters indicate significant differences between research groups, according to Fisher’s post-hoc test.

Analyzing control groups, a higher expression of *IL6ST* during the short-day photoperiod was stated (Figure 6, Table 6). During both photoperiods, treatment with LPS caused an increase in the examined gene’s expression in comparison to the control groups; however, during the short-day photoperiod, the expression was higher. Similarly, the leptin treatment caused the *IL6ST* expression to increase in both photoperiods, but during the short-day photoperiod, the expression was significantly higher. After the treatment with both experimental factors, the expression of *IL6ST* increased in both photoperiods, compared to the corresponding control and leptin-treated groups. During the short-day photoperiod, the expression in the LPS+LEP group was lower compared to that in the LPS group, while during the long days it did not differ from the LPS group. During the short-day photoperiod, the antagonistic effect on *IL6ST* expression of both factors was stated. All groups from the short-day photoperiod showed higher expressions of the examined gene, in comparison to the corresponding groups from the long-day photoperiod.

### 2.3. Gene Expression of Tumor Necrosis Factor-α and Its Receptors

**Figure 7:**
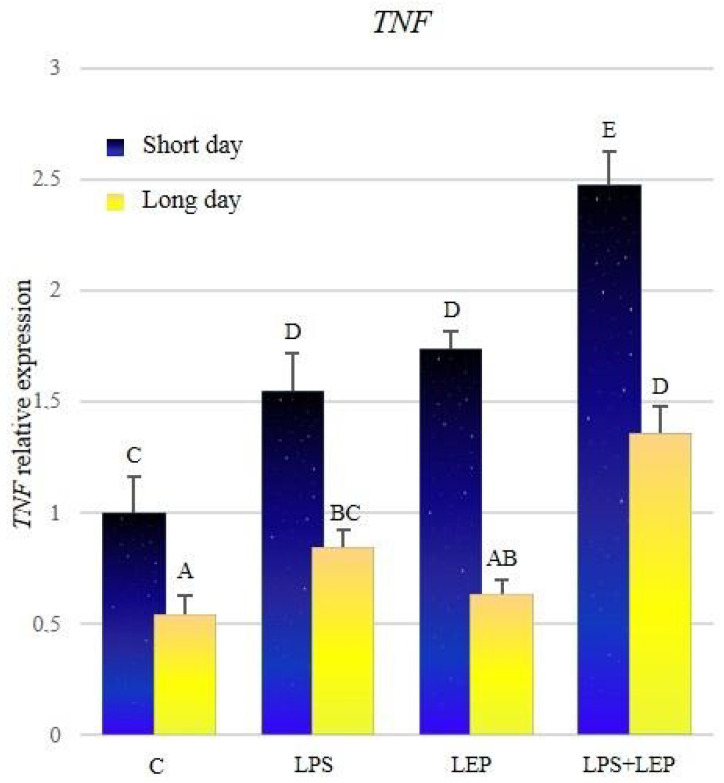
*TNF* gene expression in relation to the mean of *HDAC* and *ACTB* reference genes in the sheep’s anterior pituitary during the short-day and long-day photoperiod. Groups: C—control groups, LPS—LPS-treated groups, LEP—leptin-treated groups, LPS+LEP—groups treated with LPS and leptin. The data are presented as means ± standard error of the mean (SEM). Letters indicate significant differences between research groups, according to Fisher’s post-hoc test.

The expression of *TNF* was significantly higher during the short-day photoperiod in all research groups (Figure 7, Table 7). After the LPS treatment, the expression increased during both photoperiods in comparison to the corresponding control groups. After the leptin treatment, the expression of *TNF* increased significantly only during the short-day photoperiod; however, during both photoperiods, the expression did not differ from that observed after the LPS treatment. The groups treated with both experimental factors showed a significant increase in *TNF* expression, in comparison to corresponding groups treated with leptin or LPS alone; moreover, during both photoperiods, the synergistic effect was observed.

**Figure 8:**
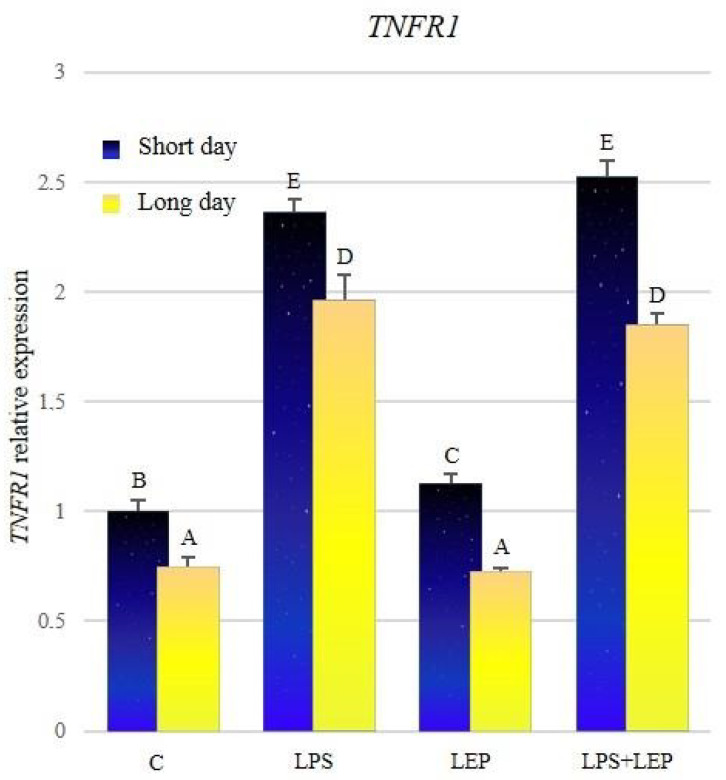
*TNFR1* gene expression in relation to the mean of *HDAC* and *ACTB* reference genes in the sheep’s anterior pituitary during the short-day and long-day photoperiod. Groups: C—control groups, LPS—LPS-treated groups, LEP—leptin-treated groups, LPS+LEP—groups treated with LPS and leptin. The data are presented as means ± standard error of the mean (SEM). Letters indicate significant differences between research groups, according to Fisher’s post-hoc test.

The expression of *TNFR1* was significantly higher in all groups from the short-day photoperiod in comparison to corresponding groups from the long-day photoperiod (Figure 8, Table 8). After the LPS treatment, the gene expression increased significantly during both photoperiods, in comparison to the corresponding control groups. Leptin treatment increased the expression of *TNFR1* in comparison to the control group only during the short-day photoperiod. Groups that received both experimental factors also showed a significant increase in the expression in comparison to the corresponding control groups; however, during both photoperiods, the expressions did not differ from those observed after the LPS treatment.

**Figure 9:**
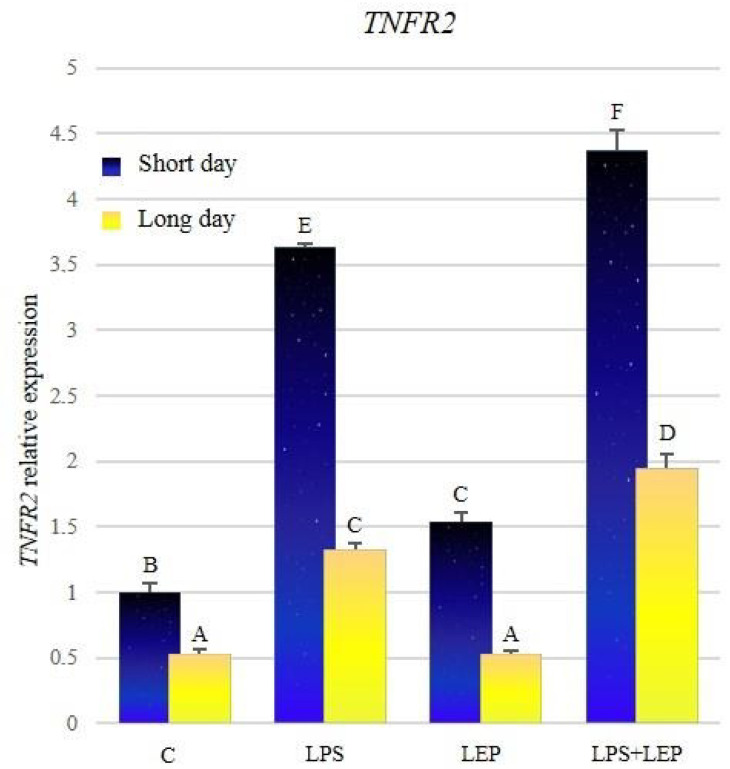
*TNFR2* gene expression in relation to the mean of *HDAC* and *ACTB* reference genes in the sheep’s anterior pituitary during the short-day and long-day photoperiod. Groups: C—control groups, LPS—LPS-treated groups, LEP—leptin-treated groups, LPS+LEP—groups treated with LPS and leptin. The data are presented as means ± standard error of the mean (SEM). Letters indicate significant differences between research groups, according to Fisher’s post-hoc test.

The expression of *TNFR2* was significantly higher in groups from the short-day photoperiod, in comparison to corresponding groups from the long-day photoperiod (Figure 9, Table 9). Both groups that received the LPS showed a significant increase in the *TNFR2* expression in comparison to the corresponding control groups. Leptin treatment caused an increase of *TNFR2* expression only during the short-day photoperiod. Groups that received both experimental factors showed a significant increase in *TNFR2* expression compared to the corresponding groups treated with leptin or LPS alone. In both LPS+LEP groups, the synergistic effect was shown.

## 3. Discussion

The current study shows that photoperiod affects the expression of the genes encoding proinflammatory cytokines and their receptors at the level of sheep AP, as compared in short- and long-day control groups. The obtained results showed that all examined genes expressions, except *IL1B* and *IL1R2*, were significantly higher during short days in comparison with long days. Such results show the modulating effect of the photoperiod on the expression of proinflammatory cytokines, and thus on inflammation. Similar studies on the effect of photoperiod on the expression of genes encoding proinflammatory cytokines and their receptors were carried out by Król et al., 2016 [20] on the level of sheep’s *pars tuberalis,* which is a part of the pituitary [20]. The significant influence of photoperiod on *TNF* expression alone, the expression of which was higher during the short days, was observed in the mentioned study.

IL1β plays a key role as a mediator of inflammation and sickness behavior [67]. The IL1 receptor family consists of type I (IL1R1) and type II (IL1R2) receptors [68]. IL1R1 is responsible for the transduction of the IL1β signal, while IL1R2 is a decoy receptor, and is considered an inhibitor of this proinflammatory cytokine action [69]. In the present study, a seasonally increased expression of *IL1R1*, with a simultaneous lack of change in *IL1R2* mRNA level, may indicate an increase in the activity of the IL1β pathway.

### 3.1. Leptin Effect on Cytokines Induction

To verify the LEPres during the long-day photoperiod, animals in both seasons were treated intravenously with leptin. The results showed that leptin treatment during short days, when sheep do not show LEPres, increased the expression of all examined genes. However, this effect was suppressed in the long days, except for *IL6R* and *IL6ST*. Such observation may suggest that the LEPres occurs at the level of AP during the long-day photoperiod. The occurrence of LEPres in the “long-day sheep” was stated earlier as happening at the level of the hypothalamus [15,16], or in the thoracic perivascular adipose tissue (PVAT) [70].

### 3.2. The Influence of Leptin on Proinflammatory Cytokines during Inflammation

In the next step of the present study, acute inflammation was induced by animal treatment with LPS, which is the most potent of all the pathogen-associated molecular pattern molecules [71]. During infection, LPS is released into the bloodstream as a result of bacterial cell lysis and replication [72]. It is worth noting that the endotoxin keeps exercising a negative influence on the host organism even after the bacteria are dead. This is because the lysis of bacteria enables the release of LPS from their cell walls, and increases the toxic effect of endotoxin [73]. LPS is widely used to induce an acute and chronic immune response in animal experiments [74]. The common structural pattern of LPS is recognized by a cascade of LPS receptors and accessory proteins: the LPS binding protein (LBP), CD14, and the Toll-like receptor4 (TLR4)–myeloid differentiation factor 2 (MD-2) complex [75]. The activation of the TLR4 complex usually leads to the secretion of inflammatory mediators, such as IL1β, IL6, or TNFα [74,76]. The results of the present study also showed an increase in the expression of all examined genes encoding the aforementioned proinflammatory cytokines, and their receptors, in sheep pituitary after the LPS intravenous injection, regardless of the examined season.

A variety of cell types secrete IL1β; however, most of the studies described the secretion by innate immune cells, such as monocytes and macrophages [77]. Many factors stimulate its expression, such as microbial stimuli (including LPS [78,79]), and other pathogens that activate pathogen recognition receptors (PRR), including TLR4 [80], other cytokines (e.g., TNFα) or IL1 by itself, and the complement protein C5a [81,82]. Krawczyńska et al., 2019 [70] stated that LPS increased IL1β expression in ovine thoracic perivascular adipose tissue during both short- and long-day photoperiods. Similar results were also shown at the level of ewe’s aorta [83]. Herman et al., 2010 also demonstrated the stimulating effect of LPS on the ovine IL1β during the anoestrous season (April–May) at the level of many structures of the hypothalamus, such as the preoptic area, the anterior hypothalamus, the medial basal hypothalamus and the medial eminence [84]. The stimulating effect of LPS on IL1β expression was also stated in the murine pituitary [85].

IL6 is involved in various autoimmune and inflammatory diseases. Its signal is transduced by two molecules: IL6R and IL6ST. IL6R has a small, 82-amino acid cytoplasmic domain, and acts as a ligand binder. The binding of IL6 to IL6R triggers the association of this complex to IL6ST. IL6ST, in turn, has an intrinsic kinase domain that contains regions required for its association with a non-receptor tyrosine kinase called JAK (Janus Kinase) [86], a region of target proteins’ phosphorylation [87]. The action of IL6ST is shared among the whole family of IL6 cytokines, and its expression is ubiquitous. In the IL6 signal transduction pathway, IL6R is more restricted [88]. It was shown that IL6 expression might be stimulated by direct LPS action from TLR-4 [89], or indirectly by TNFα and IL1β [90,91,92]. The ex vivo experiment conducted on the AP derived from sheep showed that LPS addition into a medium with explants collected from animals already treated with LPS significantly increased IL6 expression, which was not stated for explants derived from animals not treated with LPS [93]. Intracerebroventricular (icv) administration of LPS into the third ventricle increased the expression of IL6 in the ovine hypothalamic preoptic area during the reproductive season [94]. Further, IL1β injection into the third ventricular of the ovine brain caused a significant increase in *IL6* and *IL6ST* expression in both photoperiods, while *IL6R* expression increased only during the short-day period at the level of the pineal gland [95]. In turn, contrary to the results obtained in the present experiment, the studies conducted on the ovine hypothalamic preoptic area [96] did not show the influence of LPS on *IL6R* expression during short days. Similar results were also demonstrated in a study conducted on ewes exposed to prolonged immune stress (7 days), at the level of the hypothalamus in the anestrous period (April–May), in which LPS again did not influence *IL6R* expression [97]. In the aorta, expression of *IL6R* decreased only during the short-day period, and remained unchanged during long days, while *IL6* and *IL6ST* expression were increased significantly in both photoperiods after the LPS stimulation [83]. At the level of ovine thoracic perivascular adipose tissue, changes in the expressions of *IL6* and *IL6ST* were similar to those observed in the aorta, but the level of *IL6R* mRNA decreased after the stimulation with LPS in both photoperiods [70]. 

The last examined cytokine in the present study was TNFα, which is a transmembrane protein expressed on the surface of cells and cleaved by metalloprotease, which liberates a trimeric soluble cytokine. TNFα acts via two receptors, TNFR1 and TNFR2, which also form trimers that are suitable for TNFα binding. TNFR1 contains a death domain, so the induction of cell death signaling is carried out by this receptor [90,98]. Meanwhile, TNFR2 does not contain the death domain, but it is a more potent receptor for the TNFα ligand. TNFR1 expression is widespread in many cells. This receptor may be activated by both the soluble and transmembrane TNFα forms. On the other hand, *TNFR2* expression is more restricted. This receptor is expressed mainly by microglia and endothelial cells, and is preferentially activated by the transmembrane form of TNFα [99]. However, if the expression occurs, then it is much higher than the expression of *TNFR1* [100,101]. Its role is less known, and pathways activated by this receptor signal have both shared and opposing effects on TNFR1 [102]. TNFR2, as the opposite of TNFR1, may promote tissue repair and angiogenesis [102]. In ewes in the follicular phase of the estrous cycle, it was stated that the ICV injection of LPS increased *TNF* expression in the preoptic area of the hypothalamus [94]. On the other hand, a study conducted on the ovine AP explants showed no effect of LPS on *TNF* expression [93]. At the level of the hypothalamic preoptic area [96] and aorta [83], *TNFR1* expression remained unaffected after the LPS treatment during short days, while during long days a significant increase in the aorta was identified. In turn, in the AP and perivascular adipose tissue [70], *TNFR1* expression, in response to LPS treatment, increased significantly in both photoperiods. While in the case of *TNFR2* expression increased significantly in the aorta, perivascular adipose tissue, and the preoptic area of the hypothalamus during short days, in the long days, such an effect was identified only in the aorta and perivascular adipose tissue [70,83,96].

Knowing that the increase in the expression of genes encoding the proinflammatory cytokines is more pronounced during the short days, it also seems interesting to query whether photoperiod also affects the intensity of changes caused by LPS. The IL1β pathway analysis shows the more pronounced effect of LPS on *IL1B* and *IL1R2* expressions during the short-day season, and no significant differences in *IL1R1* expression between seasons. *IL6*, its receptor, and its signal transducer expressions were more strongly stimulated during the short-day photoperiod. Similarly, the expression of *TNF* and its receptors was higher during the short-day photoperiod. 

To verify the assumptions of this manuscript, that leptin can modulate the course of inflammation, the last group of animals in each season was treated with LPS followed by leptin injection after 30 min. The results showed the differences in leptin’s influence on the LPS-induced cytokines’ gene expression, depending on the photoperiod, and according to the LEPres results mentioned above. The synergistic effect of leptin and LPS, in the case of the majority of examined genes during the short-day period (except *IL6R* and *IL6ST*), was stated. This result was in line with assumptions derived from the effects of leptin alone during the no-LEPres period of short days. Therefore, during the long-day photoperiod, it was expected that the LEPres would affect the synergistic effect in an inhibitory manner, and therefore the expression results are limited to those observed in the group of animals receiving only LPS. This assumption was proved to be correct only in the case of some examined genes (*IL1R1*, *IL6, IL6ST,* and *TNFR1*). The expression of *IL6R* decreased significantly compared to its expression after LPS stimulation, which may suggest the inhibiting effect of leptin on *IL6R* expression during inflammation. Surprisingly, and against the expectation, although the stimulating effect of leptin observed during short days was suppressed during the long days, the expression of *IL1B*, *IL1R2*, *TNF*, and *TNFR2* during both photoperiods showed the synergistic effect of LPS and leptin. The maintenance of the synergistic effect during the long days may be partially explained by the enhancing effect of LPS, and especially TNFα, which may maintain or improve the leptin’s signal sensitivity [103,104,105,106]. However, it may also be caused by the different pathways of the actions of leptin and LPS. Leptin activity, which is also stimulated by LPS, is transduced by the JAK2-STAT3 pathway. In contrast, leptin may also act through different signaling pathways. As such, a different pathway was described in the case of leptin’s influence on TNFα expression. As observed, the leptin might stimulate TNFα via the pathway that is dependent on the phospholipase C Src/phospholipase D1 phosphatidic acid p70S6K/c-jun N-terminal protein kinase [107]. The other pathways that leptin might act on are the p38 and MAPK pathways [108]. Krawczyńska et al., 2019 show the synergistic effect of leptin’s and LPS’s action on *IL1B* in both the aorta and perivascular adipose tissue [70,83]. As was stated in the study conducted on mouse peritoneal macrophages, leptin also showed a synergistic effect with LPS on keratinocyte chemoattractant expression [109].

The expressions of *IL6R* and *IL6ST* were increased under the influence of leptin alone during the short days, which, in conjunction with the increased expression of *IL6,* may suggest that leptin alone increases the activity of this pathway in the ovine pituitary under physiological conditions. In turn, the effect of leptin during inflammation on the IL6 pathway is difficult to define clearly. On the one hand, the increase in *IL6* expression may suggest the stimulation of this pathway. On the other hand, a decrease in the expression of *IL6ST*, in parallel with no effect on *IL6R*, may indicate the inhibition of the signal transduction of this pathway. However, considering the wide affinity range of IL6ST [88], the decrease of its expression, as stated during short days, may not be significant in terms of the whole pathway’s activity during the short-day photoperiod. During the long-day photoperiod, leptin did not affect the LPS-induced changes in the expression of *IL6* or *IL6ST*, which may indicate a significant effect of LEPres on the signal transduction of this cytokine. The lack of any leptin effect on the IL6 pathway, in animals with induced acute inflammation, was also stated at the level of perivascular adipose tissue regardless of photoperiod [70].

## 4. Material and Methods

### 4.1. Animals

The in vivo experiments were carried out on 48 2–3 years old ewes. Experiments were conducted in two photoperiods: long-day (May/June) and short-day (November/December). In both experiments, animals were randomly divided into four groups (6 ewes in each):

- control—treated with saline in the volume equal to the experimental groups (0.9% *w*/*v* NaCl) (Baxter, Deerfield, IL, USA)
- LPS—intravenously injected with lipopolysaccharide (LPS) from *Escherichia coli* (400 ng/kg body weight); (Sigma-Aldrich, St. Louis, MO, USA)
- LEP—intravenously injected with ovine recombinant leptin (20 µg/kg body weight at a dose derived from experiments conducted on growing beef heifers [110]); [Protein Laboratories Rehovot (PLR) Ltd., Rehovot, Israel]
- LPS+LEP—intravenously injected with both LPS and leptin in the doses as mentioned above; the leptin injection was done 30 min after LPS injection.

Animals were maintained indoors in individual pens and exposed to natural daylight. The stress of social isolation was limited by visual contact. Ewes were fed a constant diet of commercial concentrates with hay, and water was available ad libitum. Estrous cycles of ewes were synchronized by the Chronogest^®^ CR (Merck Animal Health, Amsterdam, The Netherlands) method based on intra-vaginal sponges impregnated with 20 mg of a synthetic progesterone-like hormone for 14 days. After sponge removal, ewes received an intramuscular injection of 500 IU pregnant mare’s serum gonadotropin (PMSG) (Merck Animal Health, Amsterdam, The Netherlands). The experimental procedure began at 24 h after the PMSG injection. At an interval of 12 h before the start of the experiment, animals were deprived of food, and 3 h after the saline/LPS injection, animals were euthanized and decapitated to collect the anterior part of the pituitary. Tissues were frozen in the liquid nitrogen and stored at −80 °C until further analysis.

Experimental procedures were approved (authorization no. 56/2013 from 23 October 2013) by the 3rd Local Ethics Committee of the Warsaw University of Life Sciences–SGGW (Warsaw, Poland).

### 4.2. Real-Time RT-PCR

Total RNA from the anterior part of the pituitary was isolated using NucleoSpin RNA/Protein kit (Macherey-Nagel, Dueren, Germany). The quantity and the quality of the obtained RNA were verified using the spectrophotometric measurement of the optical density, using a spectrophotometer NanoDrop ND-1000 (Thermo Fisher Scientific, Waltham, MA, USA) at wavelengths of 260 nm, 280 nm and 230 nm, and electrophoresis in 1% agarose gel. Then obtained RNA was transcribed into cDNA using a reverse transcription kit Maxima™ First Strand cDNA Synthesis Kit for Real-Time Quantitative Polymerase Chain Reaction (RT-qPCR) (Thermo Fisher Scientific, Waltham, MA, USA). Subsequently, the obtained matrix was used in the real-time PCR reaction, which was performed using a FIREPol^®^ HOT EvaGreen qPCR Mix^®^ Plus kit (Solis Biodyne, Tartu, Estonia). The proper temperature profile for each gene was chosen after optimization following the standard protocol: 95 °C for 15 min for HOT FIREPol^®^ DNA Polymerase activation and 40 cycles at 95 °C for 5–10 s for denaturation, 60 °C for 10–30 s for annealing, and 72 °C for 15–30 s for the extension. The primers used for each examined gene are presented in Table 10. The PCR reactions were carried out using a thermocycler RotorGene Q (Qiagen, Germantown, MD, USA) with RotorGene Q software. The results obtained for the examined genes were normalized to the reference gene chosen from among three genes [glyceraldehyde 3-phosphate dehydrogenase (*GAPDH*), β-actin (*ACTB*) and histone deacetylase 1 (*HDAC1*)] using program NormFinder (Molecular Diagnostic Laboratory, Aarhus University Hospital, Aarhus, Denmark) for identification of the optimal normalization gene. The results are presented in arbitrary units, as the ratio of the target gene’s expression to the expression of the reference gene with a control group calculated as 1.

### 4.3. Statistics

The statistical analysis was performed with the use of STATISTICA 12 (StatSoft Polska Sp. z o. o., Cracow, Poland). Differences between groups were stated according to the three-way ANOVA with the season, LPS and leptin treatment as main factors. Each ANOVA was followed by post-hoc Fisher’s test, and considered statistically significant at *p* ≤ 0.05. Before each ANOVA was performed, its assumptions were checked: normality (Shapiro–Wilk’s test) and variance homogeneity (Levene’s test). If the ANOVA assumptions were violated, a natural logarithm was used on data. The data are presented as mean ± standard error of the mean (SEM). Synergistic and antagonistic effects occurrence was evaluated using means of the research groups, via the use of the Bliss Independence model [111,112].

## 5. Conclusions

In concluding, the results of the present study suggest that photoperiod conditions have a significant impact on the signaling pathways of the examined proinflammatory cytokines, which may be related to the increased activity of the immune system during the short-day photoperiod. Moreover, the photoperiod influences tissue’s sensitivity to leptin action, which may indicate the presence of leptin resistance during the long days. The photoperiod-dependent effects of leptin on the course of inflammation at the level of the ovine pituitary were also confirmed. The obtained results indicate the potential of the sheep model in studies on leptin resistance in the course of obesity. Moreover, it seems that this model can be used in research on hypophysitis, concerning human endocrinology and neuroendocrinology.

## Figures and Tables

**Table 1:** *IL1B* significance of differences between groups (*p*-value; Fisher’s post-hoc test). Asterisks indicate significant changes (*p* ≤ 0.05).

Groups		Short Day				Long Day			
		C	LEP	LPS	LPS+LEP	C	LEP	LPS	LPS+LEP
Short day	C		0.000164 *	0.000000 *	0.000000 *	0.503301	0.133028	0.000635 *	0.000000 *
	LEP	0.000164 *		0.000000 *	0.000000 *	0.000020 *	0.000001 *	0.653650	0.000125 *
	LPS	0.000000 *	0.000000 *		0.019915 *	0.000000 *	0.000000 *	0.000000 *	0.000000 *
	LPS+LEP	0.000000 *	0.000000 *	0.019915 *		0.000000 *	0.000000 *	0.000000 *	0.000000 *
Long day	C	0.503301	0.000020 *	0.000000 *	0.000000 *		0.395964	0.000083 *	0.000000 *
	LEP	0.133028	0.000001 *	0.000000 *	0.000000 *	0.395964		0.000005 *	0.000000 *
	LPS	0.000635 *	0.653650	0.000000 *	0.000000 *	0.000083 *	0.000005 *		0.000031 *
	LPS+LEP	0.000000 *	0.000125 *	0.000000 *	0.000000 *	0.000000 *	0.000000 *	0.000031 *	

Short day–research groups from the short-day photoperiod, Long day–research groups from the long-day photoperiod, C–control group, LEP–leptin treated group, LPS–group treated with lipopolysaccharide, LPS+LEP–group treated with lipopolysaccharide and leptin.

**Table 2:** *IL1R1* significance of differences between groups (*p*-values; Fisher’s post-hoc test). Asterisks indicate significant changes (*p* ≤ 0.05).

Groups		Short Day				Long Day			
		C	LEP	LPS	LPS+LEP	C	LEP	LPS	LPS+LEP
Short day	C		0.000000 *	0.000000 *	0.000000 *	0.000000 *	0.000013 *	0.000000 *	0.000001 *
	LEP	0.000000 *		0.716176	0.000003 *	0.000000 *	0.000000 *	0.071536	0.059186
	LPS	0.000000 *	0.716176		0.000001 *	0.000000 *	0.000000 *	0.145376	0.122910
	LPS+LEP	0.000000 *	0.000003 *	0.000001 *		0.000000 *	0.000000 *	0.000000 *	0.000000 *
Long day	C	0.000000 *	0.000000 *	0.000000 *	0.000000 *		0.271236	0.000000 *	0.000000 *
	LEP	0.000013 *	0.000000 *	0.000000 *	0.000000 *	0.271236		0.000000 *	0.000000 *
	LPS	0.000000 *	0.071536	0.145376	0.000000 *	0.000000 *	0.000000 *		0.927990
	LPS+LEP	0.000001 *	0.059186	0.122910	0.000000 *	0.000000 *	0.000000 *	0.927990	

Short day–research groups from the short-day photoperiod, Long day–research groups from the long-day photoperiod, C–control group, LEP–leptin treated group, LPS–group treated with lipopolysaccharide, LPS+LEP–group treated with lipopolysaccharide and leptin.

**Table 3:** *IL1R2* significance of differences between groups (*p*-values; Fisher’s post-hoc test). Asterisks indicate significant changes (*p* ≤ 0.05).

Groups		Short Day				Long Day			
		C	LEP	LPS	LPS+LEP	C	LEP	LPS	LPS+LEP
Short day	C		0.000001 *	0.000000 *	0.000000 *	0.590985	0.309193	0.030987 *	0.000000 *
	LEP	0.000001 *		0.000296 *	0.000000 *	0.000000 *	0.000000 *	0.001146 *	0.325885
	LPS	0.000000 *	0.000296 *		0.008222 *	0.000000 *	0.000000 *	0.000000 *	0.005016 *
	LPS+LEP	0.000000 *	0.000000 *	0.008222 *		0.000000 *	0.000000 *	0.000000 *	0.000001 *
Long day	C	0.590985	0.000000 *	0.000000 *	0.000000 *		0.628042	0.008290 *	0.000000 *
	LEP	0.309193	0.000000 *	0.000000 *	0.000000 *	0.628042		0.002241 *	0.000000 *
	LPS	0.030987 *	0.001146 *	0.000000 *	0.000000 *	0.008290 *	0.002241 *		0.000058 *
	LPS+LEP	0.000000 *	0.325885	0.005016 *	0.000001 *	0.000000 *	0.000000 *	0.000058 *	

Short day–research groups from the short-day photoperiod, Long day–research groups from the long-day photoperiod, C–control group, LEP–leptin treated group, LPS–group treated with lipopolysaccharide, LPS+LEP–group treated with lipopolysaccharide and leptin.

**Table 4:** *IL6* significance of differences between groups (*p*-values; Fisher’s post-hoc test). Asterisks indicate significant changes (*p* ≤ 0.05).

Groups		Short Day				Long Day			
		C	LEP	LPS	LPS+LEP	C	LEP	LPS	LPS+LEP
Short day	C		0.000073 *	0.000000 *	0.000000 *	0.000916 *	0.000203 *	0.000000 *	0.000000 *
	LEP	0.000073 *		0.000000 *	0.000000 *	0.000000 *	0.000000 *	0.000000 *	0.000000 *
	LPS	0.000000 *	0.000000 *		0.021568 *	0.000000 *	0.000000 *	0.000004 *	0.000000 *
	LPS+LEP	0.000000 *	0.000000 *	0.021568 *		0.000000 *	0.000000 *	0.000000 *	0.000000 *
Long day	C	0.000916 *	0.000000 *	0.000000 *	0.000000 *		0.613919	0.000000 *	0.000000 *
	LEP	0.000203 *	0.000000 *	0.000000 *	0.000000 *	0.613919		0.000000 *	0.000000 *
	LPS	0.000000 *	0.000000 *	0.000004 *	0.000000 *	0.000000 *	0.000000 *		0.460984
	LPS+LEP	0.000000 *	0.000000 *	0.000000 *	0.000000 *	0.000000 *	0.000000 *	0.460984	

Short day–research groups from the short-day photoperiod, Long day–research groups from the long-day photoperiod, C–control group, LEP–leptin treated group, LPS–group treated with lipopolysaccharide, LPS+LEP–group treated with lipopolysaccharide and leptin.

**Table 5:** *IL6R* significance of differences between groups (*p*-values; Fisher’s post-hoc test). Asterisks indicate significant changes (*p* ≤ 0.05).

Groups		Short Day				Long Day			
		C	LEP	LPS	LPS+LEP	C	LEP	LPS	LPS+LEP
Short day	C		0.021227 *	0.000839 *	0.001664 *	0.000000 *	0.000002 *	0.620013	0.009028 *
	LEP	0.021227 *		0.232119	0.335857	0.000000 *	0.000000 *	0.006067 *	0.000008 *
	LPS	0.000839 *	0.232119		0.812154	0.000000 *	0.000000 *	0.000190 *	0.000000 *
	LPS+LEP	0.001664 *	0.335857	0.812154		0.000000 *	0.000000 *	0.000390 *	0.000000 *
Long day	C	0.000000 *	0.000000 *	0.000000 *	0.000000 *		0.007271 *	0.000000 *	0.000001 *
	LEP	0.000002 *	0.000000 *	0.000000 *	0.000000 *	0.007271 *		0.000009 *	0.007181 *
	LPS	0.620013	0.006067 *	0.000190 *	0.000390 *	0.000000 *	0.000009 *		0.030371 *
	LPS+LEP	0.009028 *	0.000008 *	0.000000 *	0.000000 *	0.000001 *	0.007181 *	0.030371 *	

Short day–research groups from the short-day photoperiod, Long day–research groups from the long-day photoperiod, C–control group, LEP–leptin treated group, LPS–group treated with lipopolysaccharide, LPS+LEP–group treated with lipopolysaccharide and leptin.

**Table 6:** *IL6ST* significance of differences between groups (*p*-values; Fisher’s post-hoc test). Asterisks indicate significant changes (*p* ≤ 0.05).

Groups		Short Day				Long Day			
		C	LEP	LPS	LPS+LEP	C	LEP	LPS	LPS+LEP
Short day	C		0.001158 *	0.000000 *	0.000000 *	0.000000 *	0.000000 *	0.137910	0.003346 *
	LEP	0.001158 *		0.000001 *	0.000954 *	0.000000 *	0.000000 *	0.053906	0.706263
	LPS	0.000000 *	0.000001 *		0.029708 *	0.000000 *	0.000000 *	0.000000 *	0.000000 *
	LPS+LEP	0.000000 *	0.000954 *	0.029708 *		0.000000 *	0.000000 *	0.000002 *	0.000312 *
Long day	C	0.000000 *	0.000000 *	0.000000 *	0.000000 *		0.002213 *	0.000000 *	0.000000 *
	LEP	0.000000 *	0.000000 *	0.000000 *	0.000000 *	0.002213 *		0.000000 *	0.000000 *
	LPS	0.137910	0.053906	0.000000 *	0.000002 *	0.000000 *	0.000000 *		0.116029
	LPS+LEP	0.003346 *	0.706263	0.000000 *	0.000312 *	0.000000 *	0.000000 *	0.116029	

Short day–research groups from the short-day photoperiod, Long day–research groups from the long-day photoperiod, C–control group, LEP–leptin treated group, LPS–group treated with lipopolysaccharide, LPS+LEP–group treated with lipopolysaccharide and leptin.

**Table 7:** *TNF* significance of differences between groups (*p*-values; Fisher’s post-hoc test). Asterisks indicate significant changes (*p* ≤ 0.05).

Groups		Short Day				Long Day			
		C	LEP	LPS	LPS+LEP	C	LEP	LPS	LPS+LEP
Short day	C		0.000431 *	0.004784 *	0.000000 *	0.000203 *	0.006401 *	0.354653	0.042266 *
	LEP	0.000431 *		0.399832	0.030589 *	0.000000 *	0.000000 *	0.000024 *	0.089413
	LPS	0.004784 *	0.399832		0.003607 *	0.000000 *	0.000001 *	0.000334 *	0.378914
	LPS+LEP	0.000000 *	0.030589 *	0.003607 *		0.000000 *	0.000000 *	0.000000 *	0.000280 *
Long day	C	0.000203 *	0.000000 *	0.000000 *	0.000000 *		0.232630	0.003060 *	0.000000 *
	LEP	0.006401 *	0.000000 *	0.000001 *	0.000000 *	0.232630		0.059313	0.000013 *
	LPS	0.354653	0.000024 *	0.000334 *	0.000000 *	0.003060 *	0.059313		0.004221 *
	LPS+LEP	0.042266 *	0.089413	0.378914	0.000280 *	0.000000 *	0.000013 *	0.004221 *	

Short day–research groups from the short-day photoperiod, Long day–research groups from the long-day photoperiod, C–control group, LEP–leptin treated group, LPS–group treated with lipopolysaccharide, LPS+LEP–group treated with lipopolysaccharide and leptin.

**Table 8:** *TNFR1* significance of differences between groups (*p*-values; Fisher’s post-hoc test). Asterisks indicate significant changes (*p* ≤ 0.05).

Groups		Short Day				Long Day			
		C	LEP	LPS	LPS+LEP	C	LEP	LPS	LPS+LEP
Short day	C		0.033938 *	0.000000 *	0.000000 *	0.000009 *	0.000003 *	0.000000 *	0.000000 *
	LEP	0.033938 *		0.000000 *	0.000000 *	0.000000 *	0.000000 *	0.000000*	0.000000 *
	LPS	0.000000 *	0.000000 *		0.230573	0.000000 *	0.000000 *	0.001927 *	0.000108 *
	LPS+LEP	0.000000 *	0.000000 *	0.230573		0.000000 *	0.000000 *	0.000051*	0.000002 *
Long day	C	0.000009 *	0.000000 *	0.000000 *	0.000000*		0.723256	0.000000 *	0.000000 *
	LEP	0.000003 *	0.000000*	0.000000 *	0.000000 *	0.723256		0.000000 *	0.000000 *
	LPS	0.000000 *	0.000000 *	0.001927 *	0.000051 *	0.000000 *	0.000000 *		0.335630
	LPS+LEP	0.000000 *	0.000000 *	0.000108 *	0.000002 *	0.000000 *	0.000000 *	0.335630	

Short day–research groups from the short-day photoperiod, Long day–research groups from the long-day photoperiod, C–control group, LEP–leptin treated group, LPS–group treated with lipopolysaccharide, LPS+LEP–group treated with lipopolysaccharide and leptin.

**Table 9:** *TNFR2* significance of differences between groups (significant changes are marked with *p*-values; Fisher’s post-hoc test). Asterisks indicate significant changes (*p* ≤ 0.05).

Groups		Short Day				Long Day			
		C	LEP	LPS	LPS+LEP	C	LEP	LPS	LPS+LEP
Short day	C		0.000000 *	0.000000 *	0.000000 *	0.000000 *	0.000000 *	0.000254 *	0.000000 *
	LEP	0.000000 *		0.000000 *	0.000000 *	0.000000 *	0.000000 *	0.053487	0.002733 *
	LPS	0.000000 *	0.000000 *		0.015297 *	0.000000 *	0.000000 *	0.000000 *	0.000000 *
	LPS+LEP	0.000000 *	0.000000 *	0.015297 *		0.000000 *	0.000000 *	0.000000 *	0.000000 *
Long day	C	0.000000 *	0.000000 *	0.000000 *	0.000000 *		0.819236	0.000000 *	0.000000 *
	LEP	0.000000 *	0.000000 *	0.000000 *	0.000000 *	0.819236		0.000000 *	0.000000 *
	LPS	0.000254 *	0.053487	0.000000 *	0.000000 *	0.000000 *	0.000000 *		0.000007 *
	LPS+LEP	0.000000 *	0.002733 *	0.000000 *	0.000000 *	0.000000 *	0.000000 *	0.000007 *	

Short day–research groups from the short-day photoperiod, Long day–research groups from the long-day photoperiod, C–control group, LEP–leptin treated group, LPS–group treated with lipopolysaccharide, LPS+LEP–group treated with lipopolysaccharide and leptin.

**Table 10:** Primers used for gene expression assay.

Gene Symbol	Primer		Gene Bank, Accession Number	References
	Forward	Reverse		
*ACTB*	GCCAACCGTGAGAAGATGAC	TCCATCACGATGCCAGTG	NM_001009784.2	[83]
*HDAC1*	CTGGGGACCTACGGGATATT	GACATGACCGGCTTGAAAAT	XM_004005023.3	[97]
*GAPDH*	TGACCCCTTCATTGACCTTC	GATCTCGCTCCTGGAAGATG	NM_001190390.1	[84]
*IL1B*	CAGCCGTGCAGTCAGTAAAA	GAAGCTCATGCAGAACACCA	NM_001009465.2	[84]
*IL1R1*	GGGAAGGGTCCACCTGTAAC	ACAATGCTTTCCCCAACGTA	NM_001206735.1	[97]
*IL1R2*	CGCCAGGCATACTCAGAAA	GAGAACGTGGCAGCTTCTTT	NM_001046210.2	[83]
*IL6*	GTTCAATCAGGCGATTTGCT	CCTGCGATCTTTTCCTTCAG	NM_001009392.1	[97]
*IL6R*	TCAGCGACTCCGGAAACTAT	CCGAGGACTCCACTCACAAT	NM_001110785.3	[97]
*IL6ST*	GGCTTGCCTCCTGAAAAACC	ACTTCTCTGTTGCCCACTCAG	XM_012096909.2	[20]
*TNF*	CAAATAACAAGCCGGTAGCC	AGATGAGGTAAAGCCCGTCA	NM_001024860.1	[97]
*TNFR1* (*TNFRSF1A*)	AGGTGCCGGGATGAAATGTT	CAGAGGCTGCAGTTCAGACA	NM_001166185.1	[97]
*TNFR2* (*TNFRSF1B*)	ACCTTCTTCCTCCTCCCAAA	AGAAGCAGACCCAATGCTGT	NM_001040490.2	[97]
